# Describing the factors related to rural podiatry work and retention in the podiatry workforce: a national survey

**DOI:** 10.1186/s13047-023-00603-5

**Published:** 2023-02-07

**Authors:** Anna Couch, Hylton B. Menz, Belinda O’Sullivan, Terry Haines, Cylie M. Williams

**Affiliations:** 1 Peninsula Health, Allied Health, 2 Hastings Road, Frankston, VIC 3199 Australia; 2 Monash University, School of Primary and Allied Health Care, Moorooduc Hwy, Frankston, Australia; 3 La Trobe University, School of Allied Health, Human Services and Sport, Bundoora, VIC 3086 Australia; 4 The University of Queensland, Rural Clinical School, Toowoomba, QLD 4350 Australia; 5 Murray Primary Health Network, Bendigo, Victoria 3550 Australia; 6 Monash University School of Rural Health, Bendigo, Victoria 3550 Australia

**Keywords:** Podiatry, Rural work, Retention, Professional satisfaction, Rural training, Rural background

## Abstract

**Background:**

Maldistribution of podiatrists limits capacity to address the footcare needs of the population. Understanding factors that impact recruitment and retention of Australian podiatrists is a key solution. The primary aim of this study was to describe factors related to rural podiatry work, and overall professional retention amongst Australian podiatrists.

**Methods:**

We used data collected from the most recent relevant response of a cohort of Australian podiatrists between 2017 and 2020 of four online surveys. Person and job role variables known to impact current work and retention were collected. Logistic regression models were used to determine factors associated with rural work and intent to leave direct patient care or the profession entirely.

**Results:**

There were 1129 podiatrists (21% of 5429) who participated in at least one of the survey waves. Podiatrists who had a rural background (30%) were less likely to work in a metropolitan location (OR = 0.20, 95%CI = 0.11,0.37). Podiatrists who undertook a regional/rural placement during their undergraduate education (43%) were more likely to work in a metropolitan location (OR = 1.86, 95%CI = 1.38,2.51). Podiatrists who indicated they were planning to leave direct patient care within 5 years (*n* = 282, 26%), were less satisfied with working conditions (OR = 0.77, 95% CI = 0.66, 0.92), less satisfied with opportunities to use their abilities (OR = 0.83, 95% CI = 0.69, 0.99), perceived less personal accomplishment (OR = 0.94, 95% CI 0.86, 0.94) and less job satisfaction (OR = 0.92, 95% CI = 0.91, 0.98). Podiatrists who indicated that they were planning to leave podiatry work entirely within 5 years (*n* = 223, 21%), were less satisfied with opportunities to use their abilities (OR = 0.74, 95% CI = 0.62, 0.88), agreed they had a poor support network from other podiatrists (OR = 1.35, 95% CI = 1.13, 1.61), had less job satisfaction (OR = 0.89, 95% CI = 0.86, 0.94), and did not have access to paid annual leave (OR = 0.62, 95% CI = 0.38, 0.99).

**Conclusion:**

Findings suggest ways to promote rural work, including selecting university students with rural backgrounds, and optimising the experience of rural placements which currently predict metropolitan practice. To retain podiatrists, it is important to ensure access to leave, professional support, and appropriate physical working conditions. Further research is required to understand why intention to leave is so high.

**Supplementary Information:**

The online version contains supplementary material available at 10.1186/s13047-023-00603-5.

## Background

Globally, challenges with recruitment and retention of allied health professionals to rural and remote locations are well recognised [1, 2]. Access to healthcare should be equitable no matter where you live, however, maldistribution of healthcare workers results in urban populations having greater access [1, 3]. In Australia, people living in rural and remote areas experience higher rates of illness, hospitalisation and death when compared to other Australians [4, 5], yet these are the areas where there are greater shortages of health workers [6]. Recruiting and retaining an adequate and appropriately qualified health workforce is fundamental for the provision of high quality, comprehensive and accessible health service in rural and remote locations [7]. This may be even more complex in small, predominantly private sector allied health professions such as the Australian podiatry workforce.

Approximately 6% of the Australian podiatry workforce are located in outer regional or remote settings [8], with an estimated 16 podiatrists per 100,000 in major cities and 6 to 10 podiatrists per 100,000 in outer regional or remote settings [9]. Poorer foot health outcomes, such as higher amputation rates, have also been identified in people who live outside of urban locations [10]. Inadequate access to podiatry workforce and its services is thought to contribute to this disparity [10]. Podiatrists play a fundamental role in the provision of primary health care in different settings including acute hospitals, sub-acute rehabilitation services, aged care, private practice and with participants of the National Disability Insurance Scheme [11]. General practitioners increasingly rely on podiatrists for the management of patients with foot problems and the introduction of Medicare funding has been effective in increasing access for patients with chronic diseases to podiatry services [12].

Retaining podiatrists in the profession is critically important for optimising rural workforce supply [13], the development of strong relationships with clients [14] and enhancing health outcomes within all communities [15]. There are multiple government-funded initiatives that have been developed to improve retention of rural and remote primary healthcare workers. Broadly, these programs aim to target recognised factors seen to impact retention such as education and training, financial incentives, and professional support [13]. Whilst these initiatives exist, there has been very limited exploration of their applicability to the rural podiatry workforce.

There is a large body of research into recruitment and retention of medical and nursing professionals into rural and remote practice, but less research about allied health [16]. Allied health workforce research is challenging due to the diversity between disciplines, including different and complex funding models, education pathways and workplace structures [17]. A recent systematic review synthesized the factors impacting rural and remote allied health recruitment. These included previous rural practice exposure, tertiary scholarships, inclusion of rural content within the pre-registration education, return of service requirements and family and friends living in the same location, opportunities to progress career, access to ongoing professional development and access to mentoring/supervision [17]. Factors impacting professional retention included lack of opportunity for professional support and development, professional isolation, not having access to appropriate resources to perform the job role, and unmanageable caseloads [17]. Data relating to podiatry were embedded within overall analyses of the allied health cohort, so attributing factors specifically to the podiatry workforce has been challenging.

Therefore, the primary aim of this study was to describe factors relating to rural podiatry work, and overall professional retention amongst Australian podiatrists.

## Methods

### Design

This was a cross sectional study of Australian podiatrists using data collected through an online survey from 2017 to 2020. The CHERRIES (Checklist for Reporting Results of Internet E-Surveys) guided the reporting of collected data [18]. The Monash University Human Research Ethics Committee approved this research (19959).

### Participants and setting

Podiatrists and podiatric surgeons working in Victoria were invited to participate in Waves 1 and 2 (2017–2018) of the survey. The survey was open to all podiatrists and podiatric surgeons in Australia for Waves 3 and 4 (2019–2020). There were an estimated 5429 podiatrists and 36 podiatric surgeons registered in Australia when the final wave closed [19]. There were no restrictions on practice setting.

### Data collection

Data were collected as part of the Podiatrists in Australia: Investigating Graduate Employment (PAIGE) study, and the methodology is published [20]. PAIGE survey questions were based on a longstanding longitudinal medical workforce survey in Australia, the Medicine in Australia: Balancing Employment and Life (MABEL) study [21], tailored to exploring different elements of the podiatry workforce with a core bank of questions each wave and question bank elements added at each wave (Table 1). Data were collected relating to demographics (all waves), measurement of constructs impacting on labour decisions such as job satisfaction (all waves), earnings (Wave 1), impact on family (Wave 1), workplace setting (all waves), and mental health (Waves 2, 3 and 4). All four surveys are provided as Supplementary Files 1, 2, 3 and 4.

**Table 1:** Summary of data collected in PAIGE longitudinal surveys

Domain	Wave 1	Wave 2	Wave 3	Wave 4
Demographics	✓	✓	✓	✓
Job satisfaction	✓	✓	✓	✓
Industry lead career education and progression	✓	✓	✓	✓
Work setting	✓	✓	✓	✓
Family and Social	✓	✓	✓	✓
Finances	✓			
Discrete choice experiment on job choices	✓			
Brief Resilience Scale		✓	✓	✓
Burnout		✓	✓	✓
Personality		✓	✓	✓
Personal life events		✓	✓	✓
Mental distress		✓	✓	✓
Risk taking behaviour		✓	✓	✓
Lifelong learning attributes				✓
Social media use				✓
Coronavirus pandemic impact on practice				✓

Demographic data collected from participants included information about their gender, age, pre-registration education, postcode, current work setting and employee/employer status, number of working locations, time spent working at a location, exposure to regional/rurality placement during education, leave availability and professional development availability.

All waves of the PAIGE study included the 10-item revised job satisfaction scale [22]. Participants were asked to indicate satisfaction relating to different aspects of work. The original 7-point Likert scale used within the MABEL study was reduced to 5-point item scale (1 = *very dissatisfied*, 2 = *moderately dissatisfied*, 3 = *neutral*, 4 = *moderately satisfied*, 5 = *very satisfied*) [23]. A *not applicable* response option was also provided for each item. This adaption was on suggestion from members of the MABEL team who provided advice during survey build [24].

Waves 2, 3 and 4 included the abbreviated Maslach Burnout Inventory (aMBI), a nine-item scale used for assessing burnout [25, 26]. It has three subscales including emotional exhaustion, depersonalization and personal accomplishment [27]. An additional three questions were included on job satisfaction with reference to being a health professional [28]. Items were scored on a seven-point Likert scale *(1 = everyday, 2 = a few times a week, 3 = once a week, 4 = a few times a month, 5 = once a month or less, 6 = a few times a year, 7 = never).* Higher scores for emotional exhaustion [10–18] and depersonalisation [10–18] and lower scores for personal accomplishment (0–9) and job satisfaction (0–9) indicated greater burnout [27].

### Procedure

Podiatrists and podiatric surgeons working in Victoria (Waves 1 and 2) and Australia (Waves 3 and 4) were invited to participate in the online survey every year through its promotion on social media (Facebook, Twitter, LinkedIn and Instagram), at podiatry conferences and through targeted emails from peak bodies such as the Australian Podiatry Association and Australasian College of Podiatric Surgeons. Podiatrists who had completed previous waves and left contact details were emailed directly. Podiatrists who responded were given the opportunity to enter a competition for gift cards or to receive professional development vouchers.

Qualtrics® software (Qualtrics, Provo, UT, USA) was used to collect each wave of the online survey data [29] and subsequent waves linked responses by each participant creating their own unique identifier code. Forced or requested response prompts were used to minimise missing data, and participants could withdraw at any time by closing their internet browser. All non-completed questions were treated as missing data for the remaining non-completed variables. Question logic was used to minimise question blocks if a participant indicated that they had participated in previous waves. These logics included if there were no job changes, or no changes in location, these responses were carried over at each round. Cookies were used to allow responses to be saved up to 4 hours within partial completion. Code routinely collects Internet Protocol (IP) addresses as part of the de-identified metadata in the survey response and IPs were only viewed and used as a last resort to match data where other linking variables were incomplete.

### Analysis

**Fig. 1:**
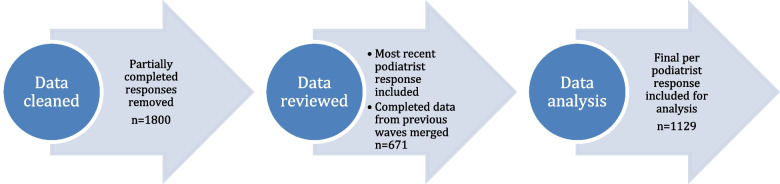
Summary of data analysis

All data were initially cleaned to remove any responses that were partially completed, including where there were no core demographics (core data included gender, age, postcode, recency of practice). As podiatrists were only requested to complete some sets when their job role or living situation had changed, a final per podiatrist response was created with the most recent response, with additional completed data from prior waves inputted as required whereby the most recent response was the one applied to the current analysis (Fig. 1). In response to the coronavirus pandemic, we undertook preliminary analysis to understand if there were distinct differences between Wave 3 (2019) and Wave 4 (2020) cohorts, particularly regarding mental health scores, burnout and any impact on job satisfaction or intent to leave the profession. We did not find significant differences in our domains of interest between waves and their impact on metropolitan or rural responses, therefore all data were analysed using the combined data set from Wave 4.

Descriptive statistics of all variables were grouped and recoded where appropriate (e.g. Satisfied responses were combined with Very Satisfied). The Modified Monash Model (MMM) was used on postcode data to recode location into metro (MMM 1) or rural (MMM 2, 3, 4, 5, 6 and 7) [30]. Univariate and multivariate regression models were used to determine factors associated with location of work (as a proxy measure of recruitment) and intent to stop seeing patients (Yes versus Unknown/No) and intent to leave the profession (Yes versus Unknown/No; as a measure of retention). Univariate logistic regression was used to explore any associations between the variable of interest (location, intent to stop seeing patients, intent to leave the profession) and those known to impact recruitment and retention to build the multiple regression model. Variables known to impact recruitment and retention were chosen based on prior studies supporting their impact on recruitment and retention, including age [3], recency of practice [3], primary work setting (private or public) and business relationship (owner or partner, salaried/contract, locum/not working and other) [31], number of working locations and time spent working at a location [32], exposure to regional/rurality through life or education [6], leave availability [3], professional development availability [33] and working in a location close to family and friends [34].

Backward stepwise multivariate regression was performed where univariate analysis revealed a value of *p* < 0.20. This analysis took form by removing the variable with the least significant fit in a stepwise procedure until all remaining variables were significant at *p* < 0.05. Regression analyses were performed using Stata 15 software (StataCorp, College Station, TX, USA).

## Results

### Participant characteristics

One thousand one hundred twenty-nine podiatrists consented to participate and completed the majority of demographic questions in at least one of the survey waves. This response rate was estimated to be 21% (1129 of 5429 podiatrists) of the profession as at 2020 [19]. Table 2 displays the summary of participants’ demographics, work setting, employment profile, regional/rural exposure according to total responses, and responses according to those working in a metropolitan or rural location. Mean (SD) age of participants were 39 [11], with no significant differences between metropolitan and rural responses. A total of 412 (42%) participants had graduated greater than 11 years ago. There were 309 (35% of 882) podiatrist responses indicating a likelihood of being impacted by burnout based on having moderate-severe scores in two or more subscales of the aMBI. There were minimal differences of burnout in all four domains between podiatrists working in metropolitan and rural locations.

**Table 2:** Participant characteristics

	Total responses	Metro responses	Rural responses
	*n* = 1129(100%)	*n* = 791 (70%)	*n* = 338 (30%)
	Mean (SD) or *Median (IQR)*	Mean (SD) or *Median (IQR)*	Mean (SD) or *Median (IQR)*
**Age** (Years)	*39(11)*	*39(11)*	*39(11)*
**Gender** (Female)	758 (69%)	483 (67%)	233 (74%)
**Recency**	***n*** **= 977**	***n*** **= 683**	***n*** **= 294**
0–5 years	339 (35%)	241 (35%)	98 (33%)
6–10 years	226 (23%)	175 (26%)	51 (18%)
> 11	412 (42%)	267 (39%)	145 (49%)
**Primary work setting**	***n*** **= 1118**	***n*** **= 784**	***n*** **= 334**
Private practice	724 (65%)	516(66%)	208(62%)
Public health service	394 (35%)	268(34%)	126(38%)
**Business relationship**	***n*** **= 1129**	***n*** **= 791**	***n*** **= 338**
Owner or partner	330 (29%)	228 (29%)	102(30%)
Salaried/Contract	753 (67%)	531 (67%)	222(53%)
Locum/Not working	14 (1%)	10 (1%)	4(1%)
Other	32 (3%)	22 (3%)	10(3%)
**Working locations**	***n*** **= 829**	***n*** **= 557**	***n*** **= 272**
1 setting	325 (39%)	220 (39%)	105 (39%)
2–3 settings	379 (46%)	250 (45%)	129 (47%)
> 3 settings	233 (28%)	162 (29%)	71 (26%)
**Time you have been working in this location (years)**	***n*** **= 1125**	***n*** **= 786**	***n*** **= 333**
	4 (1.66–6)	4 (1.25–6)	4 (2–7)
**Regional/rural placements**	***n*** **= 958**	***n*** **= 435**	***n*** **= 196**
	388 (41%)	160(37%)	71(36%)
**Grew up in a rural area**	***n*** **= 240**	***n*** **= 163**	***n*** **= 73**
	104 (43%)	54 (33%)	48 (66%)
**aMBI**^**a**^	***n*** **= 882**	***n*** **= 609**	***n*** **= 265**
Emotional exhaustion	7.4 (4.8)	7.3 (4.8)	7.5 (4.7)
Depersonalisation	3.1 (3.7)	3.1 (3.7)	3.2 (3.7)
Personal accomplishment	13.9 (4.1)	14.0 (3.9)	14.1 (4.0)
Job satisfaction	6.7 (4.2)	6.7 (4.2)	6.6 (4.1)

^a^Abbreviated Maslach Burnout Inventory

### Rural work

Podiatrists who had a rural background (MMM2–7) (30%), were less likely to work in a metropolitan location (OR = 0.20, 95% CI = 0.11, 0.37), or more likely to work rurally. Podiatrists who completed a placement in a rural setting (43%), were significantly more likely to work in a metropolitan location (OR = 1.86, 95% CI = 1.38, 2.51). There was no interaction between growing up in a rural location and undertaking a placement in a rural setting and working in a rural location.

### Retention in direct patient care and the profession

There were several significant factors relating to retention (Table 3). Podiatrists who indicated they were planning to leave direct patient care within 5 years (*n* = 282, 26%), were less satisfied with working conditions (OR = 0.77, 95% CI = 0.66, 0.92), less satisfied with opportunities to use their abilities (OR = 0.83, 95% CI = 0.69, 0.99), perceived less personal accomplishment (OR = 0.94, 95% CI 0.86, 0.94) and less job satisfaction (OR = 0.92, 95% CI = 0.91, 0.98).

**Table 3:** Factors impacting retention within the profession (leave direct patient care or leave podiatry work entirely)

	Odds Ratio	95% confidence interval (CI)	***p***
**Leave direct patient care within 5 years**			
…Satisfaction with physical working conditions	0.77	0.66–0.92	0.004
---Satisfaction with opportunities to use abilities	0.83	0.69–0.99	0.035
aMBI^a^ Personal accomplishment	0.94	0.86–0.94	< 0.001
aMBI^a^ Job Satisfaction	0.92	0.91–0.98	< 0.001
**Leave podiatry work entirely within 5 years**			
---Satisfaction with opportunities to use abilities	0.74	0.62–0.88	0.001
I have a poor support network of other podiatrists like me	1.35	1.13–1.61	0.001
aMBI^a^ Job Satisfaction	0.89	0.86–0.94	< 0.001
Annual Leave (YES)	0.62	0.38–0.99	0.010

^a^Abbreviated Maslach Burnout Inventory

Podiatrists who indicated that they were planning to leave podiatry work entirely within 5 years (*n* = 223, 21%), were less satisfied with opportunities to use their abilities (OR = 0.74, 95% CI = 0.62, 0.88), agreed they had a poor support network from other podiatrists (OR = 1.35, 95% CI = 1.13, 1.61), had less job satisfaction (OR = 0.89, 95% CI = 0.86, 0.94), and did not have access to paid annual leave (OR = 0.62, 95% CI = 0.38, 0.99).

### Job satisfaction

Overall, most podiatrists indicated that they were satisfied/very satisfied in all questions relating to job roles (Table 4). Questions with the highest satisfaction levels included freedom to choose their own method of working with 964 podiatrists (85% of 1126) satisfied/very satisfied and 915 podiatrists (85% of 1072) satisfied/very satisfied with their colleagues and fellow workers. The questions with the lowest satisfaction levels included recognition received for good work with 170 podiatrists (15% of 1112) dissatisfied/very dissatisfied, and remuneration with 236 podiatrists (21% of 1118) dissatisfied/very dissatisfied. Generally, job satisfaction levels between podiatrists working in metropolitan and rural locations were very similar, except for remuneration, with 242 (72% of 333) podiatrists in rural locations being satisfied, compared to 520 (66% of 785) podiatrists in metropolitan locations.

**Table 4:** What about my job and working location keeps me satisfied

	Total responses n (%)	Metropolitan responses n (%)	Rural responses n (%)
**Freedom to choose your own method of working**	***n*** **= 1126**	***n*** **= 789**	***n*** **= 337**
Dissatisfied	71 (6%)	49 (6%)	23 (6%)
Neutral	91 (8%)	63 (8%)	27 (8%)
Satisfied	964 (85%)	677 (86%)	288 (86%)
**Amount of variety in your work**	***n*** **= 1129**	***n*** **= 792**	***n*** **= 337**
Dissatisfied	123 (11%)	90 (11%)	33 (10%)
Neutral	93 (8%)	66 (8%)	28 (8%)
Satisfied	913 (81%)	636 (81%)	276 (82%)
**Physical working conditions**	***n*** **= 1127**	***n*** **= 790**	***n*** **= 337**
Dissatisfied	146 (13%)	105 (13%)	41 (12%)
Neutral	112 (10%)	80 (10%)	31 (9%)
Satisfied	869 (77%)	605 (77%)	265 (79%)
**Opportunities to use your abilities**	***n*** **= 1127**	***n*** **= 791**	***n*** **= 336**
Dissatisfied	120 (11%)	92 (12%)	30 (9%)
Neutral	99 (9%)	73 (9%)	26 (8%)
Satisfied	908 (80%)	626 (79%)	280 (83%)
**Your colleagues and fellow workers**	***n*** **= 1072**	***n*** **= 752**	***n*** **= 320**
Dissatisfied	70 (7%)	53 (7%)	17 (5%)
Neutral	87 (8%)	51 (7%)	36 (11%)
Satisfied	915 (85%)	648 (86%)	267 (84%)
**Recognition you get for good work**	***n*** **= 1112**	***n*** **= 779**	***n*** **= 333**
Dissatisfied	170 (15%)	124 (16%)	46 (14%)
Neutral	186 (17%)	119 (15%)	67 (20%)
Satisfied	756 (68%)	536 (69%)	220 (66%)
**Your hours of work**	***n*** **= 1125**	***n*** **= 790**	***n*** **= 335**
Dissatisfied	136 (12%)	100 (13%)	36 (11%)
Neutral	121 (11%)	80 (10%)	41 (12%)
Satisfied	868 (77%)	610 (77%)	258 (77%)
**Your remuneration**	***n*** **= 1118**	***n*** **= 785**	***n*** **= 333**
Dissatisfied	236 (21%)	181 (23%)	55 (17%)
Neutral	120 (11%)	84 (11%)	36 (11%)
Satisfied	762 (68%)	520 (66%)	242 (72%)
**Amount of responsibility you are given**	***n*** **= 1102**	***n*** **= 772**	***n*** **= 330**
Dissatisfied	74 (7%)	53 (7%)	21 (6%)
Neutral	130 (12%)	95 (12%)	35 (11%)
Satisfied	898 (81%)	624 (81%)	274 (83%)
**Taking everything into consideration, how do you feel about your job?**	***n*** **= 1128**	***n*** **= 791**	***n*** **= 337**
Dissatisfied	123 (11%)	92 (12%)	31 (9%)
Neutral	92 (8%)	63 (8%)	29 (9%)
Satisfied	913 (81%)	636 (80%)	277 (82%)
**The balance between my personal and professional commitments is about right.**	***n*** **= 1129**	***n*** **= 792**	***n*** **= 337**
Disagree	254 (22%)	183 (23%)	71 (21%)
Neutral	191 (17%)	128 (16%)	63 (19%)
Agree	684 (61%)	481 (61%)	203 (60%)
**I have a poor support network of other podiatrists like me**	***n*** **= 1121**	***n*** **= 788**	***n*** **= 333**
Disagree	634 (57%)	457 (58%)	177 (53%)
Neutral	194 (17%)	131 (17%)	63 (19%)
Agree	293 (26%)	200 (25%)	93 (28%)
**It is difficult to take time off when I want to**	***n*** **= 1122**	***n*** **= 787**	***n*** **= 335**
Disagree	538 (48%)	382 (48%)	156 (47%)
Neutral	184 (16%)	126 (16%)	58 (17%)
Agree	400 (36%)	279 (36%)	121 (36%)
**I can take time off at short notice, for example if one of my children is ill or for a home emergency**	***n*** **= 1099**	***n*** **= 766**	***n*** **= 333**
Disagree	249 (23%)	170 (22%)	79 (24%)
Neutral	174 (16%)	123 (16%)	51 (15%)
Agree	676 (61%)	473 (62%)	203 (61%)
**My patients have unrealistic expectations about how I can help them**	***n*** **= 1108**	***n*** **= 780**	***n*** **= 328**
Disagree	524 (47%)	362 (46%)	162 (50%)
Neutral	325 (30%)	225 (29%)	100 (30%)
Agree	259 (23%)	193 (25%)	66 (20%)
**The majority of my patients have complex health and social problems**	***n*** **= 1107**	***n*** **= 778**	***n*** **= 329**
Disagree	198 (18%)	146 (19%)	52 (16%)
Neutral	225 (20%)	157 (20%)	68 (20%)
Agree	684 (62%)	475 (61%)	209 (64%)
**I have good support and supervision from podiatrists with advanced skills (ie: sports, paediatrics, high risk, surgery)**	***n*** **= 1077**	***n*** **= 759**	***n*** **= 318**
Disagree	392 (36%)	260 (34%)	132 (41%)
Neutral	212 (20%)	150 (20%)	62 (19%)
Agree	473 (44%)	349 (46%)	124 (40%)
**The hours I work are unpredictable**	***n*** **= 1124**	**n = 787**	**n = 337**
Disagree	841 (75%)	583 (74%)	258 (76%)
Neutral	114 (10%)	79 (10%)	35 (11%)
Agree	169 (15%)	125 (16%)	44 (13%)
**Running my practice is stressful most of the time**	***n*** **= 767**	***n*** **= 540**	***n*** **= 227**
Disagree	282 (37%)	192 (35%)	90 (40%)
Neutral	231 (30%)	160 (30%)	71 (31%)
Agree	254 (33%)	188 (35%)	66 (29%)
**I often undertake tasks that somebody less qualified could do**	***n*** **= 1113**	***n*** **= 781**	***n*** **= 332**
Disagree	305 (27%)	218 (28%)	87 (26%)
Neutral	201 (18%)	133 (17%)	69 (21%)
Agree	607 (55%)	430 (55%)	177 (53%)
**I cannot work my preferred hours due to a lack of jobs offering those hours**	***n*** **= 1017**	***n*** **= 716**	***n*** **= 301**
Disagree	704 (69%)	489 (68%)	215 (69%)
Neutral	174 (17%)	129 (18%)	45 (15%)
Agree	139 (14%)	98 (14%)	41 (14%)
**I don’t have family members or friends in my current work location**	***n*** **= 1028**	***n*** **= 714**	***n*** **= 314**
Disagree	298 (29%)	214 (30%)	84 (27%)
Neutral	124 (12%)	89 (12%)	35 (11%)
Agree	595 (58%)	401 (56%)	194 (62%)
N/A^a^	11 (1%)	10 (1%)	1 (0%)
**My partner does not have many family members or friends in my work location**	***n*** **= 1028**	***n*** **= 714**	***n*** **= 314**
Disagree	286 (28%)	213 (30%)	73 (23%)
Neutral	110 (11%)	80 (11%)	30 (10%)
Agree	418 (41%)	276 (39%)	142 (45%)
N/A	214 (21%)	145 (20%)	69 (22%)
**There are good employment opportunities for my partner in my work location**	***n*** **= 1028**	***n*** **= 714**	***n*** **= 314**
Disagree	418 (41%)	274 (38%)	144 (46%)
Neutral	159 (15%)	114 (16%)	45 (14%)
Agree	220 (21%)	161 (22%)	59 (19%)
N/A	231 (22%)	165 (23%)	66 (21%)
**The choice of schools for our children is adequate in this work location**	***n*** **= 1028**	***n*** **= 714**	***n*** **= 314**
Disagree	445 (43%)	288 (40%)	157 (50%)
Neutral	95 (9%)	71 (10%)	24 (8%)
Agree	80 (8%)	61 (9%)	19 (6%)
N/A	408 (40%)	294 (41%)	114 (36%)

^a^Not Applicable

Job satisfaction was further explored by asking podiatrists’ levels of agreement with an additional 11 statements. Statements that podiatrists agreed with the most (defined as a response of agree or strongly agree), included the belief that most of their patients have complex, health, and social problems, with 684 podiatrists (62% of 1107) agreeing with this statement, and 676 podiatrists (61% of 1099) agreeing that they can take time off at short notice. Statements that podiatrists disagreed with most (defined as a response of disagree or strongly disagree) related to work hours, with 841 podiatrists (75% of 1124) disagreeing that the hours they work are unpredictable and 704 podiatrists (69% of 1017) disagreeing that they cannot work their preferred hours due to lack of jobs offering those hours. More than half of the podiatrists (55% of 1113) agreed that they often undertook tasks that someone less qualified could do. A total of 473 (44% of 1077) agreed that they have good support and supervision from podiatrists with advanced skills (for example sports, paediatrics, high risk, surgery), however, this was lower in rural locations (40% of 318) compared to metropolitan locations (46% of 759).

Over half of the respondents (58% of 1028) agreed that they didn’t have family or friends in their current location and 418 (41% of 1028) disagreed that there are good employment opportunities for their partner in their current work location (Table 4). This was higher (46% of 314) for participants who lived in rural locations, compared to podiatrists working in metropolitan settings (38% of 714). Exactly half (50% of 314) of podiatrists working in rural locations disagreed that the choice of schools for their children is adequate in their work location.

### Intention to leave and other work and personal factors

There were 282 (26% of 1084) podiatrists indicating they were likely/very likely to leave direct patient care within 5 years and 223 (21% of 1104) reported that they were likely/very likely to leave podiatry work entirely within the next 5 years (Table 5). When asked about hours of work, 195 (30% of 654) reported that they would like to decrease their working hours, whereas 125 (20% of 635) would like to have increased their working hours (Table 5).

**Table 5:** Intentions and opportunities

	Total responses n (%)	Metropolitan responses n (%)	Rural responses n (%)
**Intent to leave direct patient care within five years**	***n*** **= 1084**	***n*** **= 760**	***n*** **= 324**
Unlikely	683 (63%)	472 (62%)	211 (65%)
Neutral	119 (11%)	82 (11%)	37 (11%)
Likely	282 (26%)	206 (27%)	76 (23%)
**Leave podiatry work entirely within five years**	***n*** **= 1104**	***n*** **= 773**	***n*** **= 331**
Unlikely	777 (70%)	536 (69%)	241 (73%
Neutral	104 (9%)	72 (9%)	32 (10%)
Likely	223 (21%)	165 (21%)	58 (17%)
**Intent to change hours worked**	***n*** **= 635**	***n*** **= 423**	***n*** **= 212**
Desire to Increase	125 (20%)	80 (19%)	45 (21%)
	***n*** **= 654**	***n*** **= 427**	***n*** **= 227**
Desire to decrease	195 (30%)	135 (28%)	60 (26%)
**Professional development opportunities**	***n*** **= 1106**	***n*** **= 772**	***n*** **= 334**
Very Limited	118 (11%)	72 (9%)	46 (14%)
Average	458 (41%)	308 (40%)	150 (45%)
Very Good	530 (48%)	392 (50%)	138 (41%)
**Access to leave**	***n*** **= 816**	***n*** **= 577**	***n*** **= 239**
Paid annual leave	706 (87%)	471 (81%)	235 (98%)
Unpaid annual leave	521 (64%)	376 (73%)	145 (61%)
Paid sick Leave	570 (70%)	380 (72%)	190 (79%)
No Leave available	134 (16%)	93 (16%)	41 (17%)
**Opportunities for social interaction for you and your family**	***n*** **= 1042**	***n*** **= 718**	***n*** **= 318**
Very limited	162(16%)	121 (17%)	40 (13%)
Average	344 (33%)	238(33%)	105(33%)
Very Good	536 (51%)	359(50%)	173 (54%)

A total of 706 (87% of 816) of podiatrists had access to paid annual leave, 521 (64% of 816) had access to unpaid annual leave, 570 (70% of 816) had access to paid sick leave and 134 (16% of 816) had no leave available (Table 5). Overall, podiatrists working in rural locations had more access to paid annual leave (98% of 235) and paid sick leave (79% of 239) when compared to podiatrists working in metropolitan locations.

Of the total number of podiatrists, 530 (48% of 1106) reported that they had very good opportunities for continued professional development. From these responses, 392 (50% of 772) of podiatrists in metropolitan locations indicated that they had very good opportunities for continued professional development, compared to 138 (41% of 334) in rural locations. Over half, 51% of 1042) podiatrists reported they had very good opportunities for social interaction for themselves and their family.

## Discussion

This is the first national-scale study describing the rural podiatry workforce and factors related to professional retention. This substantially improves the quality of research specifically about the Australian podiatry workforce to inform policy and programs to promote rural work and professional retention. These are significant issues in maintaining overall workforce size and distribution to meet the footcare needs of the Australian population and to ensure sufficient referral options for general practitioners.

This study identified an association between working in a rural location and having a rural background, which aligns with existing high quality national-scale research about the rural medical workforce [35]. However, podiatrists who completed a rural placement were more likely to work in a metropolitan location, which contrasts with evidence from medicine and reviews of the allied health literature [17, 36]. A focus on rural placements has been a key strategy to build a well distributed high quality and sustainable health workforce [30]. The Australian Rural Health Commissioner has advised that the future allied health workforce is reliant on the growth of training and practice networks through partnerships between health services, tertiary institutions, peak bodies, and government entities [37]. This finding should be interpreted in the context of a limited sample size of students who have undertaken placements rurally, given the limited numbers of rural training placements that are available. Further, it is possible that students in podiatry have different learning expectations than current rural placements are providing, and it might be important to understand the quality of rural compared with metropolitan training posts in podiatry. There could be further analysis as to whether this effect stands for fully regionalised podiatry programs, which was beyond the scope of this paper.

Generally, job satisfaction levels between podiatrists working in metropolitan and rural locations were similar, except for podiatrists in rural locations indicating that they had less support from podiatrists with advanced skills and podiatrists in rural locations being more satisfied with their remuneration. Podiatrists who worked in rural locations indicated that they had less opportunities for continued professional development but more access to leave (paid annual and sick leave) compared to podiatrists working in rural locations. This could be addressed through advocacy of the professional association for more standardisation of employment models that are attractive and retentive.

Intent to leave direct patient care was associated with professional burnout elements, less satisfaction with physical working conditions and less satisfaction with the opportunities to use clinical abilities. This suggests the profession needs to consider developing career structure and advancing opportunities to extend skills through diversity of teaching, learning and professional clinical extension opportunities irrespective of where podiatrists practice.

Factors related to rural podiatry work from this study are similar to those impacting rural general practice and other medical specialist work through the similarly designed MABEL study [13]. The MABEL survey revealed that rural doctors had poorer professional development opportunities [38] and less access to supervision by more senior doctors [39] compared to specialists practicing in metropolitan locations. Partnerships between rural health services and peak bodies such as the Australian Podiatry Association could be beneficial to connect podiatrists working in rural locations to provide mentoring opportunities for clinicians. Increased professional support through regular check ins has been shown to enhance career development, improve morale and promotes a well-informed and motivated workforce [40].

Podiatrists working in rural locations had more access to paid annual leave and sick leave compared to metropolitan practicing podiatrists. Whilst the data did not differentiate between salaried and contracted participants, this finding may reflect different employment models in rural settings. Employment models may be specific to the Australian context where a salaried podiatrist has access to different types of paid and unpaid leave as part of their award or negotiated employment arrangement. Whereas a contracted podiatrist may work in the true sense of contractor where they take a percentage of income from the patient fee and pay for overhead costs such as a room rental fee, therefore are self-employed and have no access to paid leave provisions. Whilst rural settings may have less salaried public health service positions and more small private practice businesses, the results indicate that rural setting business models may have different ways of contracting staff which includes more access to paid leave compared to metropolitan locations. As access to leave impacted intent to leave the profession entirely, employers of podiatrists in both rural and metropolitan settings should consider how leave is accessed, structured, and planned to retain their staff.

There are several study limitations to consider when interpreting these findings. Up to date and setting-relevant workforce data is essential for government entities and healthcare planners to make appropriate recommendations and funding decisions [41]. Whilst the data provides an accurate representation of the survey participants (21% of the Australian podiatry profession), results may not be generalisable to the entire Australian podiatry profession. Despite this, our responses are one of the largest collected from Australian podiatrist and the participant demographics are similar to those reported in registration data [19]. There was also a reasonable variation noted in gender, workplace, and geographical locations of respondents across different outcome and predictor variables. As such, while this limits the ability to accurately describe the true factors impacting recruitment and retention of Australian podiatrists, there is still the ability to examine the relationships between variables, such as job satisfaction domains and burnout, as these estimates do not necessarily require representative samples [42, 43]. Given the generalised nature of the questions and wording during recruitment, it is unlikely that any one domain was subject to self-selection bias during responses.

While this research provides valuable insight into the podiatry profession, it highlights future workforce responses and research opportunities. There is limited research on burnout and mental health challenges in the podiatry profession, with only one study to date [44]. Data collected in the survey may provide valuable insight into the unique factors about the podiatry profession and why approximately 30% of respondents indicated they were at risk of burnout. Given elements of burnout were factored into intent to leave patient care, these factors should be carefully explored. Despite this research providing valuable information around factors linked to podiatrists who plan to leave the profession, intent to leave is not fully understood. Further qualitative research would be valuable to explore the perceptions of podiatrists who were actively making plans to leave, and those who had recently left the profession to retrain or work in a different industry and why. This could provide insight into what is required to both attract and retain podiatrists to continue growing the profession.

## Conclusion

This is the first national-scale study to identify factors related to the work of rural podiatrists in Australia and broader professional retention. Podiatrists identified professional burnout elements, poor connection with the podiatry workforce and feeling their scope of practice was limited as associated with a desire to leave direct patient care. These factors were similar for those that indicated that they would leave the profession all together, with addition of no access to annual leave. Findings suggest ways to promote rural work, including selecting university students with rural backgrounds, and optimising the experience of rural placements. The results further identify areas that employers of podiatrists should consider in order to retain staff in clinical care and the profession, including access to leave, professional support and physical working conditions. Podiatrists overall described a high satisfaction with many aspects of their career, and this finding could be used by peak bodies and tertiary institutions as a key benefit of working as a podiatrist. Further research is required to understand why burnout is so high in the profession and what factors influence intent to leave.

## Supplementary Information

**Additional file 1.**

**Additional file 2.**

**Additional file 3.**

**Additional file 4.**

## Data Availability

Request for further details of the data set and queries relating to data sharing arrangements may be submitted to Anna Couch (anna.couch@monash.edu). Aggregate or summarised data may be shared based on reasonable request.

## Abbreviations

PAIGE: Podiatrists in Australia: Investigating Graduate Employment
aMBI: Abbreviated Maslach Burnout Inventory
IP: Internet Protocol
