# Supplementary material for: Describing the factors related to rural podiatry work and retention in the podiatry workforce: a national survey
Source: J Foot Ankle Res. 2023 Feb 7;16:4. doi: 10.1186/s13047-023-00603-5 (PMC9903282; doi:10.1186/s13047-023-00603-5)
Supplement: Supplementary file 1 — Additional file 1. [file 13047_2023_603_MOESM1_ESM.pdf]

Default Question Block

PAIGE
Podiatrists in Australia - Investigating Graduate Employment.

You are invited to take part in this research if you are a podiatrist working in Victoria, Australia. We are interested in **EVERY** Victorian podiatrist being part of this study. Your responses are essential for this research to achieve it's aims.

Having a good work-life balance is important. This helps increase the quality of care you provide to your patients. Where you work and your choice of work setting is complex. Workplace choices can be personal and financial. These choices can impact you, your family and the health outcomes of patients.

Detailed information about these factors are essential for future workforce planning in the podiatry profession. This study is similar to others underway in Medicine and Nursing. Exploration of these factors has not been undertaken in this detail for Allied Health and particularly not in the Podiatry profession.

The information that you provide in the PAIGE study will be used to:
- Understand how to improve the morale and work satisfaction of podiatrists
- Improve the access to podiatry services for every Victorian, particularly in rural and regional areas
- Improve the evidence base informing podiatry workforce policy

This research is being undertaken by researchers at Monash University (Dr Cylie Williams, Dr Jenni White, Dr Matthew McGrail, Dr Deborah Russell, Dr Belinda O'Sullivan, Anna Couch and Prof Terry Haines) and La Trobe University (Prof Hylton Menz).

It is not expected that you will directly benefit from this research and there is no payment for being part of this research.

At the end of the survey, you may choose to enter a draw to win:
- One (1) of five (5) $100 Coles Myer gift cards.

If you have graduated in the past five (5) years, you will also be invited to join a focus group, to explore work life balance issues for new podiatry graduates in greater depth.

We will also be repeating parts of this survey in 2018 to understand how life impacts on workplace decisions, we would strongly encourage you to repeat this survey next year. The second survey will collect different information in addition to any workplace or life changes.

This survey will take between 20-40 minutes. You can complete it in your own time and it is important that the whole survey is completed. There are no questions expected to cause emotional distress or discomfort.

You can withdraw at any time by closing your Internet browser window. Being part of this study is voluntary. However, if you do consent and answer questions, anything you have answered may be used within the research. You will not be able to withdraw the answers to any questions you have answered.

If you choose to leave your contact details for any reason such as to participate gift card draw, focus group participation or so you can receive results , you can be assured that your contact details will not be subsequently linked to your survey responses.

This study has been funded by the Australian Podiatry Education and Research Foundation.

The results of this survey are confidential and you will not be asked to give any identifying information in a way that your results can be matched to you. Answers are being collected through Qualtrics and you can see the privacy policy here: https://www.qualtrics.com/privacy-statement/

Any provided details will only be viewed by the research team. The collected responses will be stored in accordance with Australian Privacy Regulations, and will be kept as a password protected data file stored on a cloud based server for 15 years. If there is future funding of this project, your responses to this survey ma be linked to your responses in future surveys. A report of this study will be submitted for publication. No individual responses will be identified in any publications

A newsletter containing key survey results and information about how results are being used to advance the podiatry

profession will be produced during this project. You have the option to provide your email address to receive this at the end of the survey. It will not be linked with your survey responses.

If you would like to contact the research team about aspects of this study or have a complaint concerning the manner in which this research is being conducted, please contact the principle investigator:

Cylie Williams: cylie.williams@monash.edu
Ph: (03) 9784 2678

OR

Should you have any concerns or complaints about the conduct of the project, you are welcome to contact:

Executive Officer, Monash University Human Research Ethics (MUHREC)
Room 111, Chancellery Building E, 24 Sports Walk, Clayton Campus
Research Office, Monash University
Tel: +61 3 9905 2052 Email: muhrec@monash.edu Fax: +61 3 9905 3831

To consent to being part of this research, please create your code to enable your answers to be linked during subsequent rounds,

To create this code, please use:
- **Your two (2) initials and two (2) digits of the date in the month of your birthday.**

e.g. Cylie Williams and birthday date in the month is 28, the code example is CW28

If you have a double surname (e.g. Smith-Peterson), only the first initial - S should be used as the code should be only 4 digits.

If you are concerned you won't remember it, please write this down as the research team will not be able to track your answers to your code.

Enter your code into the box below to enter the survey.

A) About your job satisfaction

1. Please indicate how satisfied or dissatisfied you are with each of the various aspects of your job

	Very dissatisfied	Moderately dissatisfied	Neither satisfied or dissatisfied	Moderately satisfied	Very satisfied	Not applicable
Freedom to choose your own method of working	○	○	○	○	○	○
Amount of variety in your work	○	○	○	○	○	○
Physical working conditions	○	○	○	○	○	○
Opportunities to use your abilities	○	○	○	○	○	○
Your colleagues and fellow workers	○	○	○	○	○	○
Recognition you get for good work	○	○	○	○	○	○
Your hours of work	○	○	○	○	○	○
Your remuneration	○	○	○	○	○	○
Amount of responsibility you are given	○	○	○	○	○	○
Taking everything into consideration, how do you feel about your job?	○	○	○	○	○	○

2. Please indicate the degree to which you agree or disagree with the following statements

	Strongly disagree	Disagree	Neutral	Agree	Strongly Agree	Not applicable

	Strongly disagree	Disagree	Neutral	Agree	Strongly Agree	Not applicable
The balance between my personal and professional commitments is about right	◯	◯	◯	◯	◯	◯
I have a poor support network of other podiatrists like me	◯	◯	◯	◯	◯	◯
It is difficult to take time off when I want to	◯	◯	◯	◯	◯	◯
I can take time off at short notice, for example if one of my children is ill or for a home emergency	◯	◯	◯	◯	◯	◯
My patients have unrealistic expectations about how I can help them	◯	◯	◯	◯	◯	◯
The majority of my patients have complex health and social problems	◯	◯	◯	◯	◯	◯
I have good support and supervision from podiatrists with advanced skills (ie: Sports, Paediatrics, High Risk, Surgery)	◯	◯	◯	◯	◯	◯
The hours I work are unpredictable	◯	◯	◯	◯	◯	◯
Running my practice is stressful most of the time	◯	◯	◯	◯	◯	◯
I often undertake tasks that somebody less qualified could do	◯	◯	◯	◯	◯	◯
I cannot work my preferred hours due to a lack of jobs offering those hours	◯	◯	◯	◯	◯	◯

3. Would you like to change your hours of work?

◯ No

◯ Yes, I'd like to increase my hours

◯ Yes, I'd like to decrease my hours

B) About your career development

4. Do you plan to apply for Australian Podiatry Council Career Framework in Paediatric, Sports or High Risk Foot or to the Australasian College of Podiatric Surgeons (ACPS)?

◯ Yes

◯ Unsure

◯ No, I'm already enrolled/have a place

◯ No, I have already completed my credential or I am a registered (with AHPRA) Podiatric Surgeon

◯ No

5. What year do you expect to begin?

◯ Not sure

◯ Year

6. Which of the following training courses have you considered, applied for, enrolled in or waiting to commence?

☐ Podiatric Surgery

☐ Paediatric Credential through the Australian Podiatry Council

☐ Sport Podiatry Credential through the Australian Podiatry Council

☐ High Risk Foot Credential through the Australian Podiatry Council

7. What is the likelihood that you will:

	Very unlikely	Unlikely	Neutral	Likely	Very likely	Not relevant as I only work in podiatry management or administration	Not relevant as I only work in podiatry academia	Not relevant as I have already left or never commenced a podiatry role
Leave direct patient care (private practice, community health or hospital) within FIVE YEARS?	○	○	○	○	○	○	○	○
Leave podiatry work entirely within FIVE YEARS?	○	○	○	○	○	○	○	○

C) About your work places

8. How many podiatrists work in your current main workplace? (Include yourself if applicable)

	Number
Female	
Males	
Unsure (place 0 in number column)	

9. Please nominate which of the following is the best fit for you primary workplace

- ○ Private setting with other health workers or professionals (including podiatry assistants)
- ○ Public funded setting with other health workers or professionals
- ○ Private setting with podiatrists only (excluding podiatry assistants)

9a. How many other health workers or professionals are employed at your current main workplace? (Please enter all that apply)

	Number
Other allied health professionals	
Podiatry assistants	
Medical staff (GP and/or specialists)	
Nurses	
Other (please list the worker and how many)	

10. What is your business relationship with your main workplace?

- ○ Owner or partner
- ○ Salaried employee (e.g. receive fixed annual salary and benefits with tax deducted)
- ○ Contracted employee (e.g. receive fixed payment for specified time or a % of billings before tax)
- ○ Locum
- ○ Other

11. When did you start working at your main workplace? (month and year)

- ☐ Month
- ☐ Year

12. My opportunities for continuing podiatry education and professional development are:

- ◯ Very limited
- ◯ Average
- ◯ Very good

13. What type of leave do you have access to as part of your employment arrangements (Select all that apply)

- ☐ Paid annual leave
- ☐ Unpaid annual leave
- ☐ Paid sick leave
- ☐ No leave available

14. How much leave have you taken in the past 12 months for recreational purposes? (Where you have taken paid and unpaid leave, please select both)

	Weeks
Weeks of paid recreational leave	
Weeks of unpaid recreational leave	

15. In your most recent USUAL week at work, for approximately how many HOURS did you undertake work in each of the following settings? (Include ALL of the work you do as a podiatrist)

	Hours
Private practice hours	
Community health centre or other state-run primary care organisation hours	
Public hospital hours	
Private hospital hours	
Residential/aged care health facility (nursing/residential home, hospice etc.) hours	
Tertiary education institution hours	
Other (please list setting and number of hours)	

D) About your workload

16. In question 15, you reported how many HOURS you worked in your MOST RECENT USUAL WEEK. Using this as your total, please break down how you spent these hours on the following activities? (Include ALL of the work you do as a podiatrist in ALL jobs/workplaces)

	Hours
Direct patient care hours (face-to-face, phone consultations, home visits, including any patient care with a student you are supervising)	
Indirect patient care hours (patient notes, reports, phone calls, care planning meetings)	
Education activity hours (teaching, research, continuing education)	
Practice management hours (including supervision of staff, ordering stock, advertising etc)	

	Hours
Other (please report what and how many hours	

17. In your most recent USUAL week at work, for around HOW MANY patients did you provide care? (Include new and existing patients in ALL SETTINGS—eg. hospital and private practice—procedures and telephone consultations for day time and out of hours)

	Number
Private practice	
Hospital/community health	
Home visit/ Residential Aged Care facilities	

18. Excluding emergencies or urgent needs, for how many days does a patient typically have to wait for an appointment with you or a podiatrist in your workplace:

- Less than 3 work days
- 4-7 work days
- 7-14 work days
- Greater than 15 workdays

19. For patients who attend with a Medicare chronic disease management plan, do you usually bulk bill?

- Yes
- No
- My workplace doesn't accept Medicare chronic disease management plans

20. What percentage of your usual working week involves home visits where care is provided in a person's home or residential aged care facility?

- <50%
- 50-99%
- 100%

21. How long does your average consultation last?

- <10 minutes
- 11-15 minutes
- 16-20 minutes
- 21-30 minutes
- >31 minutes

E) About your finances

The following information will be used to examine the effect of financial issues on your work–life balance.

Please remember your answers are anonymous. While we would prefer you answered all questions, you will also have the choice not to answer some of these questions.

This information will allow us to better understand how individual financial circumstances impact employment decisions.

What are your (approximate) TOTAL personal earnings from ALL of the work you do as a podiatrist?
(If possible, base this on your last personal income tax return or payslip)

Please write in ONE COLUMN where you have the most accurate information and can best remember.

22. Gross or net earnings in $ (Enter in only one)

- Annual Gross earnings (before tax)
- Fortnight Gross earnings (before tax)
- Annual Net earnings (after tax)
- Fortnight Gross earnings (after tax)
- Prefer not to answer

23. What is your total gross and net HOUSEHOLD income? (Include you and your partner's earnings, income from other business interests, dividends, interest etc.) Please write in this ONE CHOICE ONLY, where you have the most accurate information or can best remember.

- Annual Gross earnings (before tax)
- Fortnight Gross earnings (before tax)
- Annual Net earnings (after tax)
- Fortnight Net earnings (after tax)
- Prefer not to answer
- I do not have a partner

24. In addition to your income, did you receive any ongoing 'in kind' benefits or subsidies as part of your current job/s (e.g. *accommodation support, relocation assistance or car for personal use*)?

- Yes and the approximate $ value is:
- No

25. What is the total level of financial **debt** that you currently have as a result of your podiatry education and training? (Give dollar amount; include HECS debt, other debt associated with podiatry training expenses). Do not include fees you have paid, only any outstanding debt amount.

- Debt amount
- Unsure/prefer not to say
- No debt

26. Do you have any financial investment in a private podiatry practice practice?

- Yes
- No

26a. What is the total level of financial debt that you currently have from owning your practice or premises?

- Debt amount
- Unsure/prefer not to say
- No debt on my practice

26b. What is the status of your private practice for tax purposes?

- ◯ Sole trader
- ◯ Partnership
- ◯ Company
- ◯ Trust
- ◯ Don't know

27. Do you (or your employer) regularly contribute to a superannuation scheme?

- ◯ Yes
- ◯ No
- ◯ Prefer not to answer

28. Please indicate the degree to which you agree with the following statement: "Given my current financial situation and prospects, I believe I will have enough to live on when I retire".

- ◯ Strongly agree
- ◯ Agree
- ◯ Neutral
- ◯ Disagree
- ◯ Strongly disagree

29. How much (in dollars) did you pay for professional medical liability, or malpractice, insurance premiums in the last year? (If this was provided by someone else on your behalf, write 0)

- ◯ $ ______
- ◯ Unsure
- ◯ Prefer not to say

F) About your geographic location

30. How many locations do you practice at?

______

31. What is the suburb and postcode of your main podiatry workplace

- ☐ Suburb ______
- ☐ Postcode ______

32. How long have you been working in or close to this geographic location?

- ☐ Months ______
- ☐ Years ______

33. What is the suburb and postcode where you live?

- ☐ Suburb ______
- ☐ Postcode ______

34. The opportunities for social interaction for you and your family in the geographic location of your main workplace are:

- ◯ Very limited
- ◯ Average
- ◯ Very good

35. Please indicate the degree to which you agree or disagree with the following statements.

	Strongly agree	Agree	Neither agree nor disagree	Disagree	Strongly disagree	Not Applicable
I don't have many friends or family members in my current work location	◯	◯	◯	◯	◯	◯
It is easy to pursue my hobbies and leisure interests in my current work location	◯	◯	◯	◯	◯	◯
My partner does not have many friends or family members in this work location	◯	◯	◯	◯	◯	◯
There are good employment opportunities for my partner in this work location	◯	◯	◯	◯	◯	◯
The choice of schools for our children is adequate in this work location	◯	◯	◯	◯	◯	◯

36. For how many years did you live in a rural area up until the age you left secondary school?

- ◯ 0 years, did not live rurally
- ◯ Years

36a. Please indicate the town name and state of the main rural area where you lived up until school leaving age.

- ☐ Town
- ☐ State

G) About you

37. Year of Birth (Full year: i.e. 1980 not 80)

38. Gender

- ◯ Male
- ◯ Female
- ◯ Intersex

39. In what year did you complete your podiatry degree? (Full year: i.e. 1980 not 80)

40. In which country did you complete your podiatry degree?

- ◯ Australia
- ◯ New Zealand
- ◯ United Kingdom
- ◯ Other, please list

41. In general, would you say your health is:

- ◯ Excellent
- ◯ Very Good
- ◯ Good
- ◯ Fair
- ◯ Poor

H) About your family circumstances

42. Are you currently living with a partner or spouse?

- ◯ Yes
- ◯ No

42a. What is the employment status of your partner/spouse?

- ◯ Not in the labour force (e.g. caring for dependents, studying)
- ◯ Currently seeking work
- ◯ Full-time employment
- ◯ Part-time employment
- ◯ Not Applicable

43. How many dependent children do you have?

- ◯ 0
- ◯ 1
- ◯ 2
- ◯ 3
- ◯ 4
- ◯ 5 or more

43a. What are the ages of your dependent child/children? (If you have more than 5 children please list from your youngest)

	Age
Child 1	
Child 2	
Child 3	
Child 4	
Child 5	

44. Which of the following forms of childcare are you using for your youngest pre or school aged child? (Please select all that apply for your youngest child only)

- ☐ Relatives or friends
- ☐ Nannies
- ☐ Day care (childcare centre, family day care, kindergarten etc.)
- ☐ Before hours school care
- ☐ After hours school care
- ☐ No care

I) Your preferences for different types of jobs

This is the last component of the survey. You will be asked the same question 9 times with different options, please hang in there, it's very important information.

You will **NOT** be asked to repeated these choice questions in the survey next year if you remember the code you generated at the beginning of the survey. At the end of this, you will have the chance to enter the prize draw.

For the following questions:

– You are asked to state which of three jobs at three practices (A or B or C) you would choose, including the option of staying in your current job.

– Everything about the jobs you are comparing is the same, except for the characteristics shown below in each question.

	PRACTICE A	PRACTICE B	PRACTICE C
Earnings per week	No change	15% increase	15% decrease
Location	Costal town, Population <10,000	Inland town, Population <10,000	City/Regional town, Population >10,000
Hours worked per week	No change	10% increase	10% decrease
% home visits (including residential work)	0%	50%	100%

45. Which practice job would you choose?

- ◯ Practice A
- ◯ Practice B
- ◯ Practice C
- ◯ Stay at my current job

	PRACTICE A	PRACTICE B	PRACTICE C
Earnings per week	No change	15% increase	15% decrease
Location	Inland town, Population <10,000	City/Regional town, Population >10,000	Costal town, Population <10,000
Hours worked per week	10% increase	10% decrease	No change
% home visits (including residential work)	100%	0%	50%

46. Which practice job would you choose?

- ◯ Practice A
- ◯ Practice B
- ◯ Practice C
- ◯ Stay at my current job

	PRACTICE A	PRACTICE B	PRACTICE C
Earnings per week	No change	15% increase	15% decrease
Location	City/Regional town, Population >10,000	Costal town, Population <10,000	Inland town, Population <10,000
Hours worked per week	10% decrease	No change	10% increase
% home visits (including residential work)	50%	100%	0%

47. Which practice job would you choose?

- ◯ Practice A
- ◯ Practice B
- ◯ Practice C
- ◯ Stay at my current job

	PRACTICE A	PRACTICE B	PRACTICE C
Earnings per week	15% increase	15% decrease	No change
Location	Costal town, Population <10,000	Inland town, Population <10,000	City/Regional town, Population >10,000
Hours worked per week	10% increase	10% decrease	No change
% home visits (including residential work)	50%	100%	0%

48. Which practice job would you choose?

- ◯ Practice A
- ◯ Practice B
- ◯ Practice C
- ◯ Stay at my current job

	PRACTICE A	PRACTICE B	PRACTICE C
Earnings per week	15% increase	15% decrease	No change
Location	Inland town, Population <10,000	City/Regional town, Population >10,000	Costal town, Population <10,000
Hours worked per week	10% decrease	No change	10% increase
% home visits (including residential work)	0%	50%	100%

49. Which practice job would you choose?

- ◯ Practice A
- ◯ Practice B
- ◯ Practice C
- ◯ Stay at my current job

	PRACTICE A	PRACTICE B	PRACTICE C
Earnings per week	15% increase	15% decrease	No change
Location	City/Regional town, Population >10,000	Costal town, Population <10,000	Inland town, Population <10,000
Hours worked per week	No change	10% increase	10% decrease
% home visits (including residential work)	100%	0%	50%

50. Which practice job would you choose?

- Practice A
- Practice B
- Practice C
- Stay at my current job

	PRACTICE A	PRACTICE B	PRACTICE C
Earnings per week	15% decrease	No change	15% increase
Location	Costal town, Population <10,000	Inland town, Population <10,000	City/Regional town, Population >10,000
Hours worked per week	10% decrease	No change	10% increase
% home visits (including residential work)	100%	0%	50%

51. Which practice job would you choose?

- Practice A
- Practice B
- Practice C
- Stay at my current job

	PRACTICE A	PRACTICE B	PRACTICE C
Earnings per week	15% decrease	No change	15% increase
Location	Inland town, Population <10,000	City/Regional town, Population >10,000	Costal town, Population <10,000
Hours worked per week	No change	10% increase	10% decrease
% home visits (including residential work)	50%	100%	0%

52. Which practice job would you choose?

- ◯ Practice A
- ◯ Practice B
- ◯ Practice C
- ◯ Stay at my current job

	PRACTICE A	PRACTICE B	PRACTICE C
Earnings per week	15% decrease	No change	15% increase
Location	City/Regional town, Population >10,000	Costal town, Population <10,000	Inland town, Population <10,000
Hours worked per week	10% increase	10% decrease	No change
% home visits (including residential work)	0%	50%	100%

53. Which practice job would you choose?

- ◯ Practice A
- ◯ Practice B
- ◯ Practice C
- ◯ Stay at my current job

Do you have any further comments on podiatry employment in Victoria?

Block 1
