# Supplementary material for: Describing the factors related to rural podiatry work and retention in the podiatry workforce: a national survey
Source: J Foot Ankle Res. 2023 Feb 7;16:4. doi: 10.1186/s13047-023-00603-5 (PMC9903282; doi:10.1186/s13047-023-00603-5)
Supplement: Supplementary file 3 — Additional file 3. [file 13047_2023_603_MOESM3_ESM.pdf]

Default Question Block

PAIGE
Podiatrists in Australia - Investigating Graduate Employment.

There are many factors which podiatrists consider when choosing where to work. Additionally, many health professionals are confronted with burnout and their own mental health challenges. We are interested in determining how these factors apply to you and your situation.

This is the 3rd survey of the PAIGE study. Surveys 1 and 2 were conducted just in Victoria and now we are expanding to the whole of Australia for another two years.

We strongly encourage you to repeat this survey even if you completed it in 2018 to capture any changes in your work practices.

You are invited to take part in this research if you are a podiatrist working in Australia. We are interested in EVERY podiatrist working in Australia being part of this study. Your responses are essential for this research to achieve it's aims.

Detailed information about these factors are essential for future workforce planning in the podiatry profession. This study is similar to others underway in medicine and nursing. Exploration of these factors has not been undertaken in this detail for allied health, and particularly not in the podiatry profession.

The information that you provide in the PAIGE study will be used to:
- Understand how life impacts on workplace decisions
- Improve the access to podiatry services for every Australian, particularly in rural and regional areas
- Improve the evidence base informing podiatry workforce policy

This research is being undertaken by researchers at Monash University (A/Prof Cylie Williams, Anna Couch, Dr Deborah Russell, Dr Belinda O'Sullivan, and Prof Terry Haines) and La Trobe University (Prof Hylton Menz).

It is not expected that you will directly benefit from this research and there is no payment for being part of this research.

At the end of the survey you may choose to enter a draw to win:
- One (1) of ten (10) $100 vouchers for Continued Professional Development sessions or conferences delivered by the Australian Podiatry Association. You do not have to be a member of the APODA to use.

If you choose to leave your contact details to receive results, you can be assured that your contact details will not be subsequently linked to your survey responses. You will see this by the survey window opening up a new survey.

This survey will take up to 30 minutes depending on if you have completed it before. You can complete it in your own time and it is important that the whole survey is completed.

You can withdraw at any time by closing your Internet browser window. Being part of this study is voluntary. However, if you do consent and answer questions, anything you have answered may be used within the research. You will not be able to withdraw the answers to any questions you have answered.

This study has been partially funded by the Australian Podiatry Association to support a research worker, and researchers on the team are supported by fellowships and a stipend through the National Health and Medical Research Council and the Australian Government Research Training Program.

The results of this survey are confidential. You will not be asked to give any identifying information in a way that your results can be matched to you. Answers are being collected through Qualtrics and you can access the privacy policy here: https://www.qualtrics.com/privacy-statement/

Any provided information and details (in separate survey) will only be viewed by the research team. The collected responses will be stored in accordance with Australian Privacy Regulations, and will be kept as a password protected data file stored on a cloud based server for 15 years. If there is future funding of this project, your responses to this survey may be linked to your responses in future surveys. A report of this study will be submitted for publication. No individual responses will be identified in any publications.

A newsletter containing key survey results and information about how results are being used to advance the podiatry profession will be produced during this project and shared with participants and funder. You have the option to provide your email address to receive this at the end of the survey. It will not be linked with your survey responses.

If you would like to contact the research team about aspects of this study or have a complaint concerning the manner in which this research is being conducted, please contact the principal investigator:

Cylie Williams: cylie.williams@monash.edu
Ph: (03) 9784 2678

OR

Should you have any concerns or complaints about the conduct of the project, you are welcome to contact:
Executive Officer, Monash University Human Research Ethics (MUHREC) Room 111, Chancellery Building E, 24 Sports Walk, Clayton Campus Research Office, Monash University Tel: +61 3 9905 2052 Email: muhrec@monash.edu Fax: +61 3 9905 3831

If you completed the 1st or 2nd survey in 2017 or 2018 please enter the same code that you created in survey one.

If this is the first PAIGE survey you have complete and to consent to being part of this research, please create your code to enable your answers to be linked during subsequent rounds.

To create this code, please use:
- Your two (2) initials and two (2) digits of the date in the month of your birthday.

e.g. Cylie Williams and birthday date in the month is 28, the code example is CW28

If you have a double surname (e.g. Smith-Peterson), only the first initial - S should be used as the code should be only 4 digits.

1. Have you completed the Round 2 PAIGE survey

- Yes
- No
- Can't remember

2. Are you in the same job since the beginning of 2018?

- Yes
- No

A) About your job satisfaction

1. Please indicate how satisfied or dissatisfied you are with each of the various aspects of your job

	Very dissatisfied	Moderately dissatisfied	Neither satisfied or dissatisfied	Moderately satisfied	Very satisfied	Not applicable
Freedom to choose your own method of working	○	○	○	○	○	○
Amount of variety in your work	○	○	○	○	○	○
Physical working conditions	○	○	○	○	○	○
Opportunities to use your abilities	○	○	○	○	○	○
Your colleagues and fellow workers	○	○	○	○	○	○
Recognition you get for good work	○	○	○	○	○	○
Your hours of work	○	○	○	○	○	○
Your remuneration	○	○	○	○	○	○
Amount of responsibility you are given	○	○	○	○	○	○
Taking everything into consideration, how do you feel about your job?	○	○	○	○	○	○

2. Please indicate the degree to which you agree or disagree with the following statements

	Strongly disagree	Disagree	Neutral	Agree	Strongly Agree	Not applicable
The balance between my personal and professional commitments is about right	○	○	○	○	○	○

	Strongly disagree	Disagree	Neutral	Agree	Strongly Agree	Not applicable
I have a poor support network of other podiatrists like me	◯	◯	◯	◯	◯	◯
It is difficult to take time off when I want to	◯	◯	◯	◯	◯	◯
I can take time off at short notice, for example if one of my children is ill or for a home emergency	◯	◯	◯	◯	◯	◯
My patients have unrealistic expectations about how I can help them	◯	◯	◯	◯	◯	◯
The majority of my patients have complex health and social problems	◯	◯	◯	◯	◯	◯
I have good support and supervision from podiatrists with advanced skills (ie: Sports, Paediatrics, High Risk, Surgery)	◯	◯	◯	◯	◯	◯
The hours I work are unpredictable	◯	◯	◯	◯	◯	◯
Running my practice is stressful most of the time	◯	◯	◯	◯	◯	◯
I often undertake tasks that somebody less qualified could do	◯	◯	◯	◯	◯	◯
I cannot work my preferred hours due to a lack of jobs offering those hours	◯	◯	◯	◯	◯	◯

3. Would you like to change your hours of work?

- ◯ No
- ◯ Yes, I'd like to increase my hours
- ◯ Yes, I'd like to decrease my hours

B) About your career development

4. Do you plan to apply for Australian Podiatry Council Career Framework in Paediatric, Sports or High Risk Foot or to the Australasian College of Podiatric Surgeons (ACPS)?

- ◯ Yes
- ◯ Unsure
- ◯ No, I'm already enrolled/have a place
- ◯ No, I have already completed my credential or I am a registered (with AHPRA) Podiatric Surgeon
- ◯ No

2. What year do you expect to begin?

- ◯ Not sure
- ◯ Year

3. Which of the following training courses have you considered, applied for, enrolled in or waiting to commence?

- ☐ Podiatric Surgery
- ☐ Paediatric Credential through the Australian Podiatry Association
- ☐ Sport Podiatry Credential through the Australian Podiatry Association
- ☐ High Risk Foot Credential through the Australian Podiatry Association

4. What is the likelihood that you will:

	Very unlikely	Unlikely	Neutral	Likely	Very likely	Not relevant as I only work in podiatry management or administration	Not relevant as I only work in podiatry academia	Not relevant as I have already left or never commenced a podiatry role
Leave direct patient care (private practice, community health or hospital) within FIVE YEARS?	◯	◯	◯	◯	◯	◯	◯	◯
Leave podiatry work entirely within FIVE YEARS?	◯	◯	◯	◯	◯	◯	◯	◯

C) About your work places

1. How many locations do you practice at?

2. When did you start working at your main workplace? (month and year)

- ☐ Month
- ☐ Year

3. What is the suburb and postcode of your main podiatry workplace

- ☐ Suburb
- ☐ Postcode

4. How long have you been working in or close to this geographic location?

- ☐ Months
- ☐ Years

5. How many podiatrists work in your current main workplace? (Include yourself if applicable)

	Number
Female	
Males	
Unsure (place 0 in number column)	

6. What is your business relationship with your main workplace?

- ◯ Owner or partner
- ◯ Salaried employee (e.g. receive fixed annual salary and benefits with tax deducted)
- ◯ Contracted employee (e.g. receive fixed payment for specified time or a % of billings before tax)
- ◯ Locum
- ◯ Other

7. Please nominate which of the following is the best fit for you primary workplace

- ◯ Private setting with other health workers or professionals (including podiatry assistants)
- ◯ Public funded setting with other health workers or professionals
- ◯ Private setting with podiatrists only (excluding podiatry assistants)

8. How many other health workers or professionals are employed at your current main workplace? (Please enter all that apply or enter 0 into ones that have no others)

	Number
Other allied health professionals	
Podiatry assistants	
Medical staff (GP and/or specialists)	
Nurses	
Other (please list the worker and how many)	

9. My opportunities for continuing podiatry education and professional development are:

- ◯ Very limited
- ◯ Average
- ◯ Very good

10. What type of leave do you have access to as part of your employment arrangements (Select all that apply)

- ☐ Paid annual leave
- ☐ Unpaid annual leave
- ☐ Paid sick leave
- ☐ No leave available

11. How much leave have you taken in the past 12 months for recreational purposes? (Where you have taken paid and unpaid leave, please select both)

	Weeks
Weeks of paid recreational leave	
Weeks of unpaid recreational leave	

12. In your most recent USUAL week at work, for approximately how many HOURS did you undertake work in each of the following settings? (Include ALL of the work you do as a podiatrist)

	Hours
Private practice hours	
Community health centre or other state-run primary care organisation hours	
Public hospital hours	
Private hospital hours	
Residential/aged care health facility (nursing/residential home, hospice etc.) hours	
Tertiary education institution hours	
Other (please list setting and number of hours)	

13. In question 12, you reported how many HOURS you worked in your MOST RECENT USUAL WEEK. Using this as your total, please break down how you spent these hours on the following activities? (Include ALL of the work you do as a podiatrist in ALL jobs/workplaces)

	Hours
Direct patient care hours (face-to-face, phone consultations, home visits, including any patient care with a student you are supervising)	
Indirect patient care hours (patient notes, reports, phone calls, care planning meetings)	
Education activity hours (teaching, research, continuing education)	
Practice management hours (including supervision of staff, ordering stock, advertising etc)	
Other (please report what and how many hours	

14. For how many years did you live in a rural area up until the age you left secondary school?

- 0 years, did not live rurally
- Years

14a. Please indicate the town name and state of the main rural area where you lived up until school leaving age.

- Town
- State

D) About your workload

Has your workload changed since completing survey two in 2018?

- Yes
- No

D) About your workload

1. In your most recent USUAL week at work, for around HOW MANY patients did you provide care? (Include new and existing patients in ALL SETTINGS—eg. hospital and private practice—procedures and telephone consultations for day time and out of hours)
Place a 0 (zero) if you do not see any.

	Number
Private practice	
Hospital/community health	
Home visit/ Residential Aged Care facilities	

2. Excluding emergencies or urgent needs, for how many days does a patient typically have to wait for an appointment with you or a podiatrist in your workplace:

- Less than 3 work days
- 4-7 work days
- 7-14 work days

◯ Greater than 15 workdays

3. For patients who attend with a Medicare chronic disease management plan, do you usually bulk bill?

◯ Yes

◯ No

◯ My workplace doesn't accept Medicare chronic disease management plans

4. What percentage of your usual clinical load involves Medicare chronic disease management plans that are bulk billed?

◯ <25%

◯ 25-50%

◯ 51-75%

◯ >75%

Are you registered or accept patients who have NDIS funding? (If you are unsure, select no)

◯ Yes

◯ No

What percentage of your usual clinical load involves assessing or treating patients who have NDIS funding?

◯ <25%

◯ 25-50%

◯ 51-75%

◯ >75%

4a. What percentage of your usual working week involves home visits where care is provided in a person's home or residential aged care facility?

◯ <50%

◯ 50-99%

◯ 100%

5. How long does your average consultation last?

◯ <10 minutes

◯ 11-15 minutes

◯ 16-20 minutes

◯ 21-30 minutes

◯ >31 minutes

E) About your geographic location

Have you moved house/unit since completing survey two in 2018?

◯ Yes

◯ No

E) About your geographic location

1. What is the suburb and postcode where you live?

☐ Suburb

☐ Postcode

2. The opportunities for social interaction for you and your family in the geographic location of your main workplace are:

◯ Very limited

◯ Average

◯ Very good

3. Please indicate the degree to which you agree or disagree with the following statements.

	Strongly agree	Agree	Neither agree nor disagree	Disagree	Strongly disagree	Not Applicable
I don't have many friends or family members in my current work location	◯	◯	◯	◯	◯	◯
It is easy to pursue my hobbies and leisure interests in my current work location	◯	◯	◯	◯	◯	◯
My partner does not have many friends or family members in this work location	◯	◯	◯	◯	◯	◯
There are good employment opportunities for my partner in this work location	◯	◯	◯	◯	◯	◯
The choice of schools for our children is adequate in this work location	◯	◯	◯	◯	◯	◯

F) About you

1. Year of Birth (Full year: i.e. 1980 not 80)

2. Gender

◯ Male

◯ Female

◯ Intersex

◯ Prefer not to answer

3. In what year did you complete your podiatry degree? (Full year: i.e. 1980 not 80)

4. In which country did you complete your podiatry degree?

◯ Australia

◯ New Zealand

◯ United Kingdom

◯ Other, please list

5. Where did you complete your podiatry training?

◯ La Trobe University, Bundoora

◯ La Trobe University, Bendigo

◯ Western Sydney University

- Queensland University of Technology
- University of Newcastle
- Charles Sturt University, Albury-Wodonga
- University of South Australia
- Southern Cross University
- University of Western Australia
- Central Queensland University
- Other

6. Did you complete any rural or regional placements?

- Yes, Where?
- No

7. In general, would you say your health is:

- Excellent
- Very Good
- Good
- Fair
- Poor

Part B- Psychological Distress

We are interested in understanding some of the aspects about your health that may impact your work. Part B will focus on measuring impacts of distress, resilience and burnout.

Please note all data collected in Part B is confidential and you will not be asked to give any identifying information in a way that your results can be matched to you.

If any of the follow questions make you feel uncomfortable you can close the survey window. If you need further help, please call Life Line: 13 11 14 or see your general practitioner.

1. Brief Resilience Scale

	Strongly Disagree	Disagree	Neutral	Agree	Strongly Agree
I tend to bounce back quickly after hard times	○	○	○	○	○
I have a hard time making it through stressful events	○	○	○	○	○
It does not take me long to recover from a stressful event	○	○	○	○	○
It is hard for me to snap back when something bad happens	○	○	○	○	○
I usually come through difficult times with little trouble	○	○	○	○	○
I tend to take a long time to get over set-backs in my life	○	○	○	○	○

2. Allied Health professionals frequently have staff-client interaction that are based primarily around the client's current problems (psychologial, social or physical). Solutions to the client's issues may not always be obvious. Sometimes the chronic stress of working with these individuals can be emotionally draining and lead to burnout.

The Maslach Burnout Inventory is a tool used to assess Burnout. Please answer the following statements;

	Everyday	A few times a week	Once a week	A few times a month	Once a month or less	A few times a year	Never
I deal very effectively with the problems of my patients	○	○	○	○	○	○	○

	Everyday	A few times a week	Once a week	A few times a month	Once a month or less	A few times a year	Never
I feel I treat some patients as if they were impersonal objects	○	○	○	○	○	○	○
I feel emotionally drained from my work	○	○	○	○	○	○	○
I feel fatigued when I get up in the morning and have to face another day on the job	○	○	○	○	○	○	○
I've become more callous towards people since I took this job	○	○	○	○	○	○	○
I feel I'm positively influencing other people's lives through my work	○	○	○	○	○	○	○
Working with people all day is really a strain for me	○	○	○	○	○	○	○
I don't really care what happens to some patients	○	○	○	○	○	○	○
I feel exhilarated after working closely with my patients	○	○	○	○	○	○	○
I think of giving up podiatry for another career	○	○	○	○	○	○	○
I reflect on the satisfaction I get from being a podiatrist	○	○	○	○	○	○	○
I regret my decision to become a podiatrist	○	○	○	○	○	○	○

3. The Ten-Item Personality Inventory (TIPI)

I see myself as:

	Disagree strongly	Disagree moderately	Disagree a little	Neither agree nor disagree	Agree a little	Agree moderately	Agree strongly
1. Extraverted, enthusiastic	○	○	○	○	○	○	○
2. Critical, quarrelsome	○	○	○	○	○	○	○
3. Dependable, self-disciplined	○	○	○	○	○	○	○
4. Anxious, easily upset	○	○	○	○	○	○	○
5. Open to new experiences, complex	○	○	○	○	○	○	○
6. Reserved, quiet	○	○	○	○	○	○	○
7. Sympathetic, warm	○	○	○	○	○	○	○
8. Disorganised, careless	○	○	○	○	○	○	○
9. Calm, emotionally stable	○	○	○	○	○	○	○
10. Conventional, uncreative	○	○	○	○	○	○	○

4. The personal life events listed below can have an important influence on person's work-life balance.

For each statement below, please indicate 'YES' or 'NO' as to whether you experienced the event during the past 12 months. For each statement you answer 'YES', please indicate how long ago the event occurred or commenced.

	No	Yes	0-3 months ago	4 to 6 months ago	7 to 9 months ago	10 to 12 months ago
Serious personal injury or illness to self	☐	☐	☐	☐	☐	☐
Serious personal injury or illness to a close relative or family member	☐	☐	☐	☐	☐	☐
Death of spouse or child	☐	☐	☐	☐	☐	☐
Death of other close relative or family member (e.g parent or sibling)	☐	☐	☐	☐	☐	☐
Death of a close friend	☐	☐	☐	☐	☐	☐
Victim of physical violence (e.g assault)	☐	☐	☐	☐	☐	☐
Victim of property crime (e.g theft, housebreaking	☐	☐	☐	☐	☐	☐

	No	Yes	0-3 months ago	4 to 6 months ago	7 to 9 months ago	10 to 12 months ago
Named as defendant in a medical negligence claim	☐	☐	☐	☐	☐	☐

5. This question asks about everyday risk-taking in relation to different types of activities.

How likely are you to engage in each of the following activities?

	1. Very unlikely	2. Unlikely	3. Neutral	4. Likely	5. Very likely
Financial risks (e.g investments with an uncertain outcome)	○	○	○	○	○
Career and professional risks (e.g. publicly challenging your professional colleagues)	○	○	○	○	○
Clinical risks (e.g recommending a treatment which is new to your usual practice or is controversial)	○	○	○	○	○

6. These questions concern how you have been feeling over the past 30 days. Tick a box below each question that best represents how you have been.

	1. None of the time	2. A little of the time	3. Some of the time	4. Most of the time	5. All of the time
During the last 30 days, about how often did you feel tired out for no good reason?	○	○	○	○	○
During the last 30 days, about how often did you feel nervous?	○	○	○	○	○
During the last 30 days, about how often did you feel so nervous that nothing could calm you down?	○	○	○	○	○
During the last 30 days, about how often did you feel hopeless?	○	○	○	○	○
During the last 30 days, about how often did you feel restless or fidgety?	○	○	○	○	○
During the last 30 days, about how often did you feel so restless you could not sit still?	○	○	○	○	○
During the last 30 days, about how often did you feel depressed?	○	○	○	○	○
During the last 30 days, about how often did you feel that everything was an effort?	○	○	○	○	○
During the last 30 days, about how often did you feel so sad that nothing could cheer you up?	○	○	○	○	○
During the last 30 days, about how often did you feel worthless?	○	○	○	○	○

MONASH University

Peninsula Health

LA TROBE UNIVERSITY

Block 1
